# Pharmacogenomic profile of actionable molecular variants related to drugs commonly used in anesthesia: WES analysis reveals new mutations

**DOI:** 10.3389/fphar.2023.1047854

**Published:** 2023-03-20

**Authors:** Juan Fernando Parada-Márquez, Nicolás David Maldonado-Rodriguez, Paula Triana-Fonseca, Nora Constanza Contreras-Bravo, Carlos Alberto Calderón-Ospina, Carlos M. Restrepo, Adrien Morel, Oscar Javier Ortega-Recalde, Daniel Felipe Silgado-Guzmán, Mariana Angulo-Aguado, Dora Janeth Fonseca-Mendoza

**Affiliations:** ^1^ Department of Molecular Diagnosis, Genética Molecular de Colombia SAS, Bogotá, Colombia; ^2^ School of Medicine and Health Sciences, Center for Research in Genetics and Genomics (CIGGUR), Institute of Translational Medicine (IMT), Universidad Del Rosario, Bogotá, Colombia

**Keywords:** next-generation sequencing, pharmacogenomics, allelic frequency, molecular variants, anesthesia drugs

## Abstract

**Background:** Genetic interindividual variability is associated with adverse drug reactions (ADRs) and affects the response to common drugs used in anesthesia. Despite their importance, these variants remain largely underexplored in Latin-American countries. This study describes rare and common variants found in genes related to metabolism of analgesic and anaesthetic drug in the Colombian population.

**Methods:** We conducted a study that included 625 Colombian healthy individuals. We generated a subset of 14 genes implicated in metabolic pathways of common medications used in anesthesia and assessed them by whole-exome sequencing (WES). Variants were filtered using two pipelines: A) novel or rare (minor allele frequency—MAF <1%) variants including missense, loss-of-function (LoF, e.g., frameshift, nonsense), and splice site variants with potential deleterious effect and B) clinically validated variants described in the PharmGKB (categories 1, 2 and 3) and/or ClinVar databases. For rare and novel missense variants, we applied an optimized prediction framework (OPF) to assess the functional impact of pharmacogenetic variants. Allelic, genotypic frequencies and Hardy-Weinberg equilibrium were calculated. We compare our allelic frequencies with these from populations described in the gnomAD database.

**Results:** Our study identified 148 molecular variants potentially related to variability in the therapeutic response to 14 drugs commonly used in anesthesiology. 83.1% of them correspond to rare and novel missense variants classified as pathogenic according to the pharmacogenetic optimized prediction framework, 5.4% were loss-of-function (LoF), 2.7% led to potential splicing alterations and 8.8% were assigned as actionable or informative pharmacogenetic variants. Novel variants were confirmed by Sanger sequencing. Allelic frequency comparison showed that the Colombian population has a unique pharmacogenomic profile for anesthesia drugs with some allele frequencies different from other populations.

**Conclusion:** Our results demonstrated high allelic heterogeneity among the analyzed sampled, enriched by rare (91.2%) variants in pharmacogenes related to common drugs used in anesthesia. The clinical implications of these results highlight the importance of implementation of next-generation sequencing data into pharmacogenomic approaches and personalized medicine.

## Introduction

Interindividual variability in the efficacy of analgesic and anesthesia drugs is determined by multiple factors including gender, age, drug interactions, and molecular variants in genes related to pharmacokinetics and pharmacodynamics. This variability has been associated with adverse drug reactions (ADRs) related to morbidity and mortality worldwide (Saba et al., 2017). The incidence of serious ADRs to anesthetic drugs has been estimated at 1 per 3,000 individuals with a mortality rate of 10% (Patton and Borshoff, 2018). Among these reactions, malignant hyperthermia (MH) is one of the most severe, defined as a pharmacogenetic disorder that occurs in response to medications used during general anesthesia in susceptible patients (Rosenberg et al., 2015). This clinical condition is characterized by a hypercatabolic state, increased temperature, hypoxemia, metabolic acidosis, rhabdomyolysis and in some cases leads to death (Hopkins et al., 2021). This severe ADR has been associated with molecular variants in the *RYR1* gene (Ryanodine receptor 1) caused by gain of function alleles and increased sensitivity to channel activation (Carpenter et al., 2009b). Other drugs used in anesthesiology, such as opioids and intravenous anesthetics (e.g., propofol) have been associated with ADRs linked to molecular variants in genes such as *COMT, ABCB1, OPRM1, CYP2B6, CYP3A4, CYP2D6* (Nerenz and Tsongalis, 2018). Remarkably, about 59% of the drugs used in anesthesia clinical practice are metabolized by at least one actionable pharmacogenetic enzyme (Bugada et al., 2020; Malki and Pearson, 2020).

To date, the evaluation of single nucleotide variants (SNVs), is the most widely used molecular strategy in pharmacogenetic practice (van der Lee et al., 2020). Despite its usefulness, a considerable fraction of drug response cannot be attributed to SNVs, suggesting that rare variants in pharmacogenes are important contributors to genetic variability (Searle and Hopkins, 2009). In this context, next-generation sequencing (NGS) methods represent powerful tools to identify rare SNVs in an efficient and large-scale manner (Ingelman-Sundberg et al., 2018). Furthermore, characterization of population pharmacogenomic profiles may be useful to guide clinical interventions that improve anesthetic drugs safety and effectiveness. Latin-American populations are commonly underrepresented in pharmacogenetic studies, making clinically relevant to study such populations through large-scale genomic analysis. To identify genetic variants potentially related to pharmacological response to analgesics and anesthetic drugs in a healthy sample of Colombian individuals, we performed whole-exome sequencing (WES) and *in silico* analysis that included the identification of rare and validated genomic variants in a subset of 14 candidate genes. Our study identified 148 molecular variants potentially related to variability in the therapeutic response to 14 drugs commonly used in anesthesiology.

## Materials and methods

### Study population

We conducted an analytical observational study enrolling 625 individuals from the Colombian population without reported adverse reaction and/or therapeutic failure to drugs commonly used in anesthesia. Blood samples were obtained in a private laboratory (Genética Molecular de Colombia SAS, Bogotá- Colombia). All individuals signed an informed consent form to participate in the present study in addition to the sampling authorization for molecular and cytogenetic studies used in the laboratory under guidelines ISO 17025-2015. 509 individuals from our sample (78%) were enrolled from a previous study described by Silgado-Guzmán et al. (2022).The sample size was calculated considering the estimation of a proportion (N = [z2 *p * (1 - p)/e2]/[1 + (z2 *p * (1 - p)/(e2 *N)) with a confidence level of 95% (α: 0.05, z: 1.96), p (sample proportion) 16% and e (margin of error) 3% (Kadam and Bhalerao, 2010): where N (population size) was set at 574 (we included 625 individuals to final analysis).

Considering that there are no previous studies investigating allele frequencies of the genes included in the designed panel for our population, the value of sample proportion (16%) was estimated according to the allelic frequency related with anesthesia drugs (*CACNA1S* and *RYR1*) described by Lanillos et al., 2022 which included pharmacogenetic analysis in 5001 spanish or Latin America individuals (Lanillos et al., 2022).

This study was approved by the ethics committee of Universidad del Rosario (Approval number DVO005 1289-CV1281). The study was conducted according to the Helsinki declaration guidelines.

### Molecular and bioinformatic analysis

To identify molecular variations in genes related to anesthetic drugs response, we generated a subset of 14 genes selected according to their pharmacodynamic and pharmacokinetic relation with common drugs used in anesthesia practice (Table 1). The identification of genes related to drug metabolism (targets, enzymes, and carriers) was obtained from PharmGKB (PharmGKB, 2022), DrugBank (Drugbank, 2022) and clinical evidence documented in the literature. WES analysis and data quality control filtering was conducted as previously described by Silgado-Guzmán et al. (2022). We applied a downstream bioinformatics analysis by using two analysis filters: A) novel or rare (minor allele frequency - MAF less than 1%) variants including missense, loss-of-function (LoF, e.g., frameshift and nonsense), and splice site variants with potential deleterious effect and B) clinically validated genomic variants described in the PharmGKB (levels of evidence 1, 2 and 3) and/or ClinVar databases (Hewett et al., 2002; Landrum et al., 2020). All variants identified by filtering strategy B were considered. For filtering strategy A we included LoF (nonsense, frameshift and splicing) and missense variants with potential pathogenicity. Potential functional impact of splice site variants was assessed using adaptative boosting (ADA) and random forest (RF) scores (cutoff >/ = 0.6). For missense variants, pathogenicity was determined according to a optimized prediction framework (OPF) for pharmacogenetic genes (Zhou et al., 2019). We implemented this approach in order to study drug absorption, distribution, metabolism and excretion (ADME) genes variation in the Colombian population (Silgado-Guzmán et al., 2022). As described previously, individuals scores for LTR, MutationAssesor, PROVEAN, VEST3 and CADD algorithms were computed using ANNOVAR software. Thresholds were set according to those described by Zhou et al. (2019) and assigned as 1 (deleterious) or 0 (functionally neutral). Finally, a composite score was obtained averaging optimized individual predictions (0 or 1) (Silgado-Guzmán et al., 2022). Variants with a composite score ≥0.6 were classified as functionally relevant.

**TABLE 1:** Anesthesia gene panel for WES analysis.

Drug class	Drug	Pharmacogene
Analgesic	Fentanyl	*ABCB1*
		*CACNA1S*
		*CYP3A4*
		*COMT*
		*KCNJ6*
		*OPRM1*
		*RHBDF2*
Antiemetic prophylaxis	Ondansetron	*ABCB1*
		*CYP2D6*
	Midazolam	*CYP3A4*
		*CYP3A5*
		*POR*
Inductor	Propofol	*ABCB1*
		*ADRB2*
		*CYP2B6*
	Ketamine	*CYP2B6*
Inhaled anesthetic	Sevoflurane	*ABCB1*
		*CACNA1S*
		*RYR1*
	Isoflurane	*CACNA1S*
		*RYR1*
	Desflurane	*CACNA1S*
		*RYR1*
Local anesthetic	Bupivacaine	*CYP1A2*
Muscle relaxers	Succinylcholine	*RYR1*
	Rocuronium	*ABCB1*
Opioids	Dihydrocodeine	*CYP2D6*
	Oxycodone	*COMT*
		*CYP2D6*

### Sanger sequencing to confirm novel rare variants

Novel rare molecular variants identified by filtering strategy A were confirmed using Sanger sequencing. Briefly, genomic regions flanking variants *ABCB1* c.1323G>C; *ADRB2* c.313T>G; *CACNA1S* c.4211C>T, c.4010T>C, c.3095G>C; *CYP2B6* c.353G>A, c.648delG; *OPRM1* c.145C>T; *POR* c.2014T>G, c.1398 + 1G>C; *RHBDF2* c.1849G>A, c.1763T>C; and *RYR1* c.573C>A, c.2362G>A, c.9563C>G and c.10127A>T, were amplified using polymerase chain reaction (PCR). The primers amplification sequences and the PCR products length are described in Supplementary Table S1. The PCR products were analysed on agarose gels (1.5%) by ethidium bromide staining and sequenced. The reference sequences were obtained from the Ensembl database (Supplementary Table S1). Sequences were analysed with FinchTV v1.5.0 (Geozpiza Inc.) and compared to the wild-type version using ClustalW v2.1 (http://www.clustal.org/). For *OPRM1,* c. 310G>A Sanger validation was not possible due to insufficient DNA.

### Cloning for multiple nucleotide variant (MNV) analysis

One potential multiple nucleotide variant (MNV) in *RYR1* (changes in positions c.10126G>T and c.10127A>T) was identified by Sanger sequencing analysis (Supplementary Figure S1A). To obtain haplotype phasing, we amplified by PCR the genomic region flanking *RYR1* c.10126G>T and c.10127A>T variants and cloned into the pCR™4-TOPO™Vector according to manufacturer´s recommendations. (https://www.thermofisher.com/). The constructs were sequenced to verify the distribution of variants between both alleles (Supplementary Figures S1B and S1C).

### Population genetics analysis

Allelic, genotypic frequencies and Hardy-Weinberg equilibrium (HWE) were calculated using the SNPstats tool (Solé et al., 2006). HWE deviation was estimated using *X2* goodness-of-fit test with 1° of freedom. Pearson’s chi-square test with Yates and Bonferroni´s corrections was used to compare allelic frequencies between our study population and data from Latin-American and global populations described in the gnomAD v2.1 public database. Statistical significance was set at *p* < 0.05.

Ancestry analysis was performed using the R package EthSEQ v2.1.4. The approach was designed to produce reliable ethnicity analyses from whole exome sequencing individual´s data (Romanel et al., 2017). We integrated EthSEQ into our WES processing and used the “SS2.All.gsd” pre-computed reference model to infer ethnicity for each individual. This model was built using the 1000 genome project genotype data which included individuals from European (EUR), African (AFR), South Asian (SAS), East Asian (EAS), and mixed American populations (AMR) (Romanel et al., 2017).

## Results

### Demographic characteristics

Characteristics of subjects are summarized in Table 2. Among the 625 Colombian individuals enrolled in this study, demographic characteristics were obtained from 582 of them. 48% of the population were male (n = 282) and 52% were female (n = 300). 509 individuals from our sample (78%) were enrolled from a previous study described.

**TABLE 2:** Demographic characteristics.

	n	%
Sex		
Female	300	52
Male	282	48
Geographic región^a^		
Andean	503	86,43
Caribbean	55	9,45
Orinoquian	10	1,72
Amazonian	11	1,89
Pacific	1	0,17

^a^ Geographic region was determined according to Paredes et al., 2003.

### Molecular analysis

Results were obtained following the bioinformatic pipeline described in the methodology. A total of 148 genetic variants were identified in the 14 genes assessed. 135 (91.2%) variants were obtained with filtering strategy A and 13 (8.8%) with filtering strategy B (Supplementary Tables 2 and 3). 83.1% (123/148) correspond to rare and novel missense variants, classified as pathogenic according to a pharmacogenetic OPF (threshold ≥0.6), 5.4% (8/148) were LoF, 2.7% (4/148) led to potential splicing alterations and 8.8% (13/148) were assigned as actionable or informative pharmacogenetic variants according to PharmGKB and ClinVar drug database. 67.6% (100/148) variants were identified in pharmacodynamic genes (*ADRB2*, *CACNAS1*, *COMT*, *KCNJ6*, *OPRM1*, *POR*, *RHBDF2*, and *RYR1*), 23.6% (35/148) in phase 1 pharmacokinetic metabolism genes (*CYP1A2*, *CYP2B6*, *CYP2D6*, *CYP3A4*, and *CYP3A5*), and 8.8% (13/148) in the *ABCB1* transporter.

### Novel and rare molecular missense and LoF variants (filtering strategy A)

Pathogenic rare and novel missense variants were identified in 92.8% (13/14) of the genes included (with exception of *KCNJ6*). Using a functional prediction framework optimized for pharmacogenetics assessments, we obtained 124 missense variants predicted as pathogenic (Supplementary Table 2).13.7% (17/124) of them are not previously reported in databases. Sanger sequencing validated 14 of them: one false positive was identified in *RYR1*, c.573C>A and *OPRM1* c.310G>A validation was not possible (insufficient DNA), due to its adequate depth of coverage (35x), this variant was included in the final analysis. Finally, a multiple nucleotide variant (MNV) located in *RYR1* c.10126G>T and c.10127A>T was identified (Supplementary Figure S1A). Our cloning approach allowed us to define that MNV in *RYR1* corresponds to c.10126_10127 delinsTT, p. Glu3376Leu (Supplementary Figures S1B and S1C). Wakeling et al. (2020).

For the final analysis, 16 variants were defined as novel in the population: 14 confirmed by sanger sequencing, one MNV in *RYR1* and one with high quality of sequence data (*OPRM1* c.310G>A) (Supplementary Table 2).

*RYR1, CACNAS1S*, and *ABCB1* were highly polymorphic genes with 34.1% (42/123), 12.2% (15/123), and 9.7% (12/123) pathogenic rare or novel missense variants respectively (Supplementary Table 2).

Regarding rare LoF and splice site variants, 12 genetic variants were identified. Of these, 2 were novel and not previously described in databases: *POR* c.1398 + 1G>C and *CYP2B6* c.648delG (Supplementary Table 2). Both variants were validated by Sanger sequencing.

### Validated/actionable genomic variants (filtering strategy B)

Using filtering strategy B, we identified 13 variants with evidence levels 1, 2, and 3 according to PharmGKB and ClinVar public databases. 10 of these affected six genes involved in pharmacodynamic processes (*COMT*, *KCNJ6*, *OPRM1*, and *POR*), and 3 were in the phase 1 metabolism gene *CYP3A5*. 38.5% (5/13) were missense variants, 7.7% (1/13) LoF, 15.4% (2/13) were in the 3′UTR region, 7.7% (1/13) in the promoter region, and 30.8% (4/13) were synonymous variants. A list of the variants obtained by this filtering strategy is presented in Supplementary Table 3.

### Population genetic analysis

Our results suggest allelic frequency variability and statistically significant differences between the Colombian population and other Latin-American and global populations described in the GnomAD database (Supplementary Tables 2 and 3). 96.6% of the identified variants were in HWE (*p* > 0.05), while *COMT* p.His62 = , *ADRB2* p.Ser220Cys, *RYR1* p.Thr2787Ser, *CYP2D6* p.Tyr355Cys and *CYP3A5* p.Lys208= exhibited HWE deviation (*p* < 0.05). Comparison with Latin-American and global population allelic frequencies revealed statistically significant differences (*p* < 0.05) in 8 missense variants clinically validated and 51 rare variants (Supplementary Tables S2 and S3). Interestingly, 46.1% (6/13) of molecular variants clinically validated exhibited significantly higher frequencies in our population compared to Latin-American allelic frequencies (rs4818, rs10264272, rs2070995, rs2070995, rs1799972 and rs1057868). Of these, CYP3A5*6 (p.Lys208 = ) related with an LoF allele by splicing defect is 3.3 times more frequent in Colombian population, Our analysis demonstrated that 43.1% (60/123) of pathogenic rare missense, LoF and splice site molecular variants (Supplementary Table 2) found in this study are located in *RYR1* and *CACNAS1* genes, which are related to the malignant hyperthermia phenotype.

### Ancestry analysis

**FIGURE 1:**
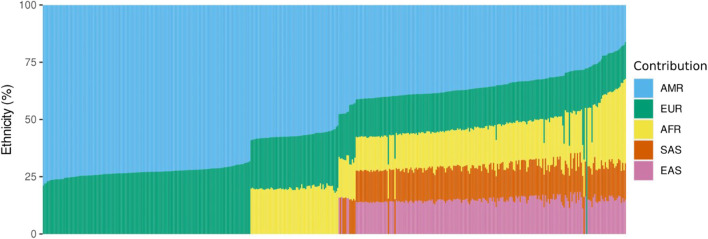
Ethnicity analysis using whole exome sequencing data. The vertical axis represents the percentage of contribution for each ethnicity, and the horizontal axis represents the individuals analyzed. AMR, American; EUR, European; AFR, African; SAS, South Asian; EAS, East Asian.

Ethnicity analysis using EthSEQ was possible for 438 samples (70%) and showed that our sampled population is highly admixed. American ethnicity was the most prevalent, with 410 individuals labelled as closest to AMR (93.6%), meaning the top-ranked contribution was mixed American population. Twenty-three individuals (5.3%) were classified as closest to African, whereas five individuals (1.1%) were inferred as closest to European population. Taking together all the population genetics data, the major ethnic contribution was American (51.7%), followed by European (22.1%), African (12.3%), South Asia (7%) and East Asia (6.9%) (Figure 1).

## Discussion

Current evidence has highlighted the importance of clinical surveillance during anesthetics administration to patients carrying altered alleles in genes related to interindividual variation to drug response (Aroke and Dungan, 2016). Latin-American countries constitute understudied populations for variants in these genes. Molecular analysis based on next-generation sequencing (NGS) techniques is an opportunity to provide data of pharmacogenetic actionable alleles and rare/novel relevant variants which facilities the integration of genetic variability in a precision medicine framework (Zhou et al., 2018).

The genetic panel selected in this study included the genes with the greatest impact on the response to anesthesia drugs. Our result indicated that 91.2% (135/148) of identified variants in this study were rare and 10.8% (16/148) of them novel, these findings are consistent with recent reports that support the usefulness of NGS to detect novel and rare variants in clinically relevant pharmacogenes (Lanillos et al., 2022). NGS pipelines are known to have random and systematic errors in sequencing, alignment and variant calling. Thus, the American College of Medical Genetics and Genomics (ACMG) recommends confirmation using Sanger sequencing (De Cario et al., 2020; Holt et al., 2021). In our analysis, 1 out of 17 filtered variants was a NGS false positive result (*RYR1* c.573C>A). Despite that in our NGS analysis average mapping efficiency was >99%, with sequencing depth on targets and coverage of target regions >90x and >99.4% respectively, *RYR1*c.573C>A display a depth coverage of 8x. Inconsistent results of Sanger sequencing can be attributed to this quality parameter. Zheng et al. (2019) proposed that a quality threshold of >35x depth coverage achieves 100% NGS true-positive (Zheng et al., 2019), highlighting the importance of quality controls.

Our approach, based on exome sequencing, is common in routine clinical genetic diagnosis and the utilization of large-scale NGS sequencing data for pharmacogenetics purposes has been reported in anesthesia. This approach has been useful to identify novel and rare variants in families affected with malignant hyperthermia, a pharmacogenetic disorder of muscle metabolism triggered by certain anesthetics such as succinylcholine (Kim et al., 2013; Riazi et al., 2018).

Additionally, exome sequencing has been successful identifying rare variants associated in individuals with disorders associated with local anesthetic resistance (Clendenen et al., 2016). At the population level, WES provides an opportunity to recover pharmacogenetic information and enables fast and cost-efficient sequencing useful for the determination of molecular profiles in populations understudied like Latin American.

A high percentage of rare pathogenic variants were identified on *CACNA1S* and *RYR1* genes, with 12.6% and 31.8%, respectively. Human linkage studies led to the discovery that mutations in both genes were involved in MH, a pharmacogenetic disorder with locus and allelic heterogeneity. In patients undergoing general anesthesia the incidence of MH has been estimated to be 0.004%–0.1%(Carpenter et al., 2009b). This receptor is critical for the activation-contraction process in myocytes by releasing calcium from the sarcoplasmic reticulum to the cytosol (Treves et al., 2008). In our population all variants in *RYR1* gene are potentially associated with MH susceptibility were heterozygous, in agreement with other reports, and probably these changes are related with gain-of-function mutations, making abnormal RYR1 channels more sensitive to activation (Riazi et al., 2018). Pathogenicity determination by an optimized prediction framework for pharmacogenetic genes supports the potential deleterious effect of the variants identified. An individual carrying MH pathogenic variant could be considered MH susceptible with increased risk of generating the disorder under anesthesia (Riazi et al., 2018).

Some of the *RYR1* variants have been functionally characterized, for instance, Ramachadran and others, identified that the pore-lininig helix region (aminoacids 4912–4948) is required for controlling RYR gating (Ramachandran et al., 2013). Considering these observations, we propose that *RYR1* p.Val4925Ile has a negative effect of the on channel gating. On the other hand, protein stability depends on residues located in repetitive regions 1-2 where we identified the variant *RYR1* p.Arg999His, potentially leading to receptor instability (Yuchi et al., 2015). We identified 3 rare variants, p.Glu653Gly, p.Val788Met and p.Gln1002Arg located in the SPRY1, repetitive region 1-2 and SPRY3 domains, respectively. Mutations affecting the SPRY1 domain could reduce the binding of FKBP (FK506-binding proteins) (Chen and Kudryashev, 2020). The variants reported in our study likely affect FKBP binding, resulting in overactivation of the receptor, potentially increasing the susceptibility to MH. The missense variant p.Gln1002Arg might affect the salt bridge between Glu917 and Arg1000, leading to an alteration of the protein´s secondary structure (Brandom et al., 2013; Chen and Kudryashev, 2020). A MNV was identified in the *RYR1* gene, this variant involved two SNVs located next to each other in the same strand of DNA (c.10126G>T and c.10127A>T). Previous studies identified that replication error introduced by DNA polymerase zeta (pol-zeta) produces MNVs, affecting mainly TC->AA, GC->AA and their reverse complements (Harris and Nielsen, 2014). Despite NGS pipeline annotating this MNV as two independent SNVs, p.Glu3376Ter and p.Glu3376Val respectively, our cloning approach determined it as p.Glu3376Leu which corresponds to rescued nonsense category (nonsense + missense: missense) (Supplementary Figure S1A) (Wang et al., 2020a). This finding highlights the importance to determine haplotype phasing to correct misannotated MNVs (Wei et al., 2015). OPF is designed to analyze separate SNVs, therefore the molecular consequence of *RYR1* MNV (p. Glu3376Leu) couldn’t be determined using this approach. It is recognized that MNVs are an important source of molecular variability, nonetheless, their functional impact and pathogenicity prediction are still unexplored (Kaplanis et al., 2019). Acknowledging this limitation, we propose that RYR1 (p.Glu3376Leu) can be considered as probably damaging using the prediction obtained from Poplyphen-2 (score 0.999) (http://genetics.bwh.harvard.edu/pph2/).

Recently, it has been recognized that in NGS analyses, MNVs can be miscalled resulting in misannotations and incorrect aminoacid prediction by GATK variant calling. Considering the negative impact on clinical care, novel NGS variant callers that incorporate haplotype information and performs phasing of SNVs have been recommended (Wang et al., 2020b; Srinivasan et al., 2021).

Despite the functional evidence being clear for some *RYR1* mutations, a large number of variants identified by WES required pathogenic assessments using bioinformatics prediction and variant interpretation software tools (Schiemann and Stowell, 2016). However, given the possibility of potential false-positives, false-negatives and incomplete penetrance, *RYR1* rare missense variants should be taken with caution.

Our bioinformatic pipeline enabled us to identify validated genomic variants which represented 8.8% (13/148). Our population displayed higher allelic frequencies compared to other Latin American populations, in five variants with known clinical pharmacogenomic effect (Supplementary Table 3). Some of these variants have been related to anesthesia drug response and up to 124 clinical annotations/anesthesic drugs pairs have been reported in PharmGKB database (https://www.pharmgkb.org/). Interestingly, we found a 3.3 times fold increase of *COMT* p. Lys208= in our population. This variant is associated with impaired fentanyl pharmacokinetics leading to decreased clearance and increased plasma concentration related to opioid toxicity (Kuip et al., 2017; Williams et al., 2022).

In addition to the implication in toxicity, genetic variants in *COMT* have been related to pain modulation, which determines the impact of these gene polymorphisms on the response to anesthetic procedures (Nackley et al., 2007). Previous reports have showed that *COMT* p.Val158Met (rs4680) decreases the enzymatic activity and patients with these polymorphism require higher doses of opioids to achieve the desired therapeutic effect (Chidambaran et al., 2012). Allelic frequency for *COMT* p.Val158Met in our population was 36%, meaning that a high percentage of Colombian patients could require dose adjustment for commonly used analgesics. Actionable synonym variants in *COMT* gene associated with therapeutic failure at conventional doses were found at high frequency in our population (COMT p.His62= and p.Leu136 = ). Recent studies indicate that patients with these variants had a higher score on the Face, Legs, Activity, Cry, Consolability (FLACC) Behavioral Pain Scale and required more frequent opioid administration compared to patients carrying wild-type alleles (Sadhasivam et al., 2014; Hooten et al., 2019). Variants in the *OPRM1* (p.Ala99Val, c.*9C>T) and *KCNJ6* (p.Pro165 = ) genes decrease opioid sensitivity of the *mu* receptor, leading to higher morphine dose requirement, generating ADRs. Tohyama and Suzuki, (1989); Nishizawa et al. (2009); Chidambaran et al. (2012). According to the frequency of these variants in our population, 84.5% of patients could be exposed to drug toxicity attributed to these polymorphisms. On a practical level, our results suggest that a significant percentage of the Colombian population is carrier of actionable pharmacogenetic variants in anesthesiology.

Another consequence related to polymorphisms in pharmacogenes is a decreased metabolic rate for common anesthetics, for example, the POR p.Ala503Val polymorphism associated to the metabolism of midazolam was found in 34% of the population. This polymorphism has been linked to an increase in the free drug fraction involved in respiratory depression and cardiovascular instability (Chidambaran et al., 2012). Similarly, the incidence of severe ADRs such as fatal arrhythmias has been associated with polymorphisms in *CYP3A5* (e.g., c.*14T>C, pThr346Tyrfs* and p.Lys208 = ), secondary to altered metabolism of drugs such as remifentanil, fentanyl, alfentanil, sufentanil, and ondansetron (Chidambaran et al., 2012; Elens et al., 2013).

Our results demonstrate high allelic heterogeneity enriched mainly by pathogenic rare and novel missense variants in anesthesia pharmacogenes. The allelic frequencies in our population compared with those reported in public databases indicate statistically significant differences. Our analysis revealed that the *RYR1* gene exhibited the highest mutational heterogeneity and some variants, such as p.Arg999His and p.Val4925Ile, occur more frequently in our population (p< 0.05) with values up to 27 times higher. The clinical implication of these findings relies on the potential incidence in our population of one of the most severe and lethal adverse effects related to anesthetic drug use, MH. A Previous study in the Caucasian population has reported a 0.05% prevalence of high-risk alleles in *RYR1* (Carpenter et al., 2009a). This value is significantly lower compared with our population, where the prevalence of rare likely pathogenic and pathogenic variants was 2.8%. Our results highlight the relevance to study and filling gaps in Latin-American populations, little studied to date in pharmacogenomics (Rodrigues-Soares et al., 2020)

Ethnicity analysis using WES data confirmed that our population is highly admixed, with 93.6% individuals labelled as closest to mixed American population. Previous ancestry studies have found similar results, underscoring the diversity of Colombian population (Ossa et al., 2016; Chande et al., 2021). A recent study by Chande et al*.* (2021), using whole-genome genotyping showed evidence of admixture with at least two major ancestry components: Native American ancestry ranging between 1.3% and 73.6% and European ancestry from 2.5% to 92.5% (Chande et al., 2021). Similarly, Ossa et al (2016) used ancestry informative markers (AIMs) to characterize the ancestry of Colombian population, finding that for the Andean region, the geographical area where most of the samples come from, the major contributions were European (58%) and Native American (35%) (Ossa et al., 2016). Similar results were obtained by Naranjo-Galvis et al. (2018) who conducted a study related to genetic polymorphisms in cytokine genes in Colombian patients with ocular toxoplasmosis. This study involved a genetic ancestries analysis using ancestry-informative insertion-deletion markers (AIM-INDELs) and interestingly, the ancestry information for the Central West Andean region of Colombia demonstrated a relevant European contribution to the genetic background for this population (Naranjo-Galvis et al., 2018). Our approach based on ancestry determination using WES individual data demonstrated a relevant admixture.

Admixture patterns on pharmacogenes variants could be important determinants of drug response. Previous studies reported that Latin-American populations have a higher frequency of ancestry-enriched SNPs when compared to ancestral populations. These differences may impact drug metabolism based on specific genetic ancestry profiles (Norris et al., 2018).

In clinical setting advances in pharmacogenetic research have contributed to the field of anesthesia, through the identification of genetic variations in drug enzymes, transport proteins, and receptors. Molecular changes have the potential to affect the pharmacokinetics and pharmacodynamics of commonly used drugs in this field, ultimately modifying their efficacy and generating potential life-threatening conditions (Saba et al., 2017). Multiple evidence supports the clinical implications of genetic variants in these genes. For example, it was shown that children with ultra-rapid metabolizers of *CYP2D6* had fatal or life-threatening events due to the administration of codeine as analgesia after adenotonsillectomy (Kelly et al., 2012). Similarly, polymorphisms in the *OPRM1* gene have been associated to postoperative pain (Lee et al., 2016). Another example of how pharmacogenomics can improve clinical outcomes in perioperative settings is the prevention of malignant hyperthermia (Hopkins et al., 2021). These studies exemplify the importance of pharmacogenomics in anesthesia, and its potential applications in personalized treatment, improving patient comfort and safety, and reducing associated morbidity and mortality (Behrooz, 2015).

### Study limitations

Through WES, we cannot identify genetic variants located in regulatory regions not contemplated in an exome sequencing analysis. Second, pathogenicity determined by *in silico* analysis should be validated by functional studies. Finally, second-generation sequencing used in NGS analysis did not allow us to identify common haplotypes, copy number variants, and structural variants for *CYP2D6*. It is recommended to apply long-read sequencing such as Oxford Nanopore Technology for *CYP2D6* analysis.

## Conclusion

Our study demonstrated the potential of NGS to identify novel and rare variants in pharmacogenes related to drugs used in anesthesia. While actual recommendations concerning clinical implementation involved only common SNVs to guide therapeutic management, the application of an optimized prediction framework for pharmacogenetic genes is useful to assess other types of potentially pathogenic variants. The incorporation of pharmacogenomic data obtained through WES analysis constitutes a valuable tool in safe and effective clinical decisions for specific populations.

## Data Availability

The original contributions presented in the study are included in the article/Supplementary Material, further inquiries can be directed to the corresponding authors.

## Supplementary material

SUPPLEMENTARY FIGURE S1 Multiple-nucleotide variant (MNV) in *RYR1* gene. **(A)**. Sanger sequencing chromatogram showing the *RYR1* c. 1026G>T and c.1027A>T genotypes that identified a multiple nucleotide variant (MNV). **(B)**. Haplotype phasing obtained by cloning approach showing allele wild type. **(C)**. Haplotype phasing obtained by cloning approach showing allele carrier of MNV.

## Abbreviations

**ACMG**, American college of medical genetics and genomics; **ADA**, Adaptative boosting; **ADRs**, adverse drug reactions; **apoCaM**, apo calmodulin; **BWT**, Burrows-Wheeler; **DHPR**, domain and dihydropyridine receptor; **DHPR**, domain and dihydropyridine receptor; **FATHMM**, functional analysis through hidden Markov models; **FLACC**, Face, Legs, Activity, Cry, Consolability scale; **gnomAD**, genome aggregation database; **HSF**, Human Splicing Finder; **HWE**, Hardy-Weinberg equilibrium; **LoF**, loss-of-function; **MAF**, minor allele frequency; **MH**, malignant hyperthermia; **NGS**, next-generation sequencing; **OPF**, optimized prediction framework; **PharmGKB**, pharmacogenomics knowledge base; **RF**, random forest; **SIFT**, sorting intolerant from tolerant; **SNV**, single nucleotide variants; Transformation; **GATK**, Genome Analysis toolkit; **WES**, whole exome sequencing; **MNV**, Multiple-nucleotide variant.
